# Assessment of laypersons’ paediatric basic life support and foreign body airway obstruction management skills: a validity study

**DOI:** 10.1186/s13049-018-0544-8

**Published:** 2018-09-06

**Authors:** Asbjørn Hasselager, Doris Østergaard, Tim Kristensen, Claus Sønderskov, Cathrine Bohnstedt, Torsten L. B. Lauritsen, Lars Konge, Martin G. Tolsgaard

**Affiliations:** 1 Copenhagen Academy for Medical Education and Simulation (CAMES), Herlev Ringvej 75, 2730 Herlev, Denmark; 2 University of Copenhagen, Nørregade 10, 101 Copenhagen, Denmark; 3 Department of Children and Adolescence Medicine, Herlev Hospital, Herlev Ringvej 75, 2730 Herlev, Denmark; 4 RedMitBarn – FirstAiders, Rosenørns Alle 1, 1970 Frederiksberg C, Denmark; 5 Department of paediatrics, North Zealand Hospital, Dyrehavevej 29, 3400 Hillerød, Denmark; 6 Department of Anaesthesia, The Juliane Marie Centre, Rigshospitalet University Hospital, Blegdamsvej 9, 2100 Copenhagen, Denmark; 7 Copenhagen Academy for Medical Education and Simulation (CAMES), Blegdamsvej 9, 2100 Copenhagen, Denmark

**Keywords:** Paediatric basic life support, Paediatric CPR, Laypersons, Foreign body airway obstruction, Training, Assessment, PBLS, FBAO, Validity, Pass/fail

## Abstract

**Background:**

Standardised courses for laypeople in Paediatric Basic Life Support (PBLS) and Foreign Body Airway Obstruction Management (FBAOM) teach essential skills for the initiation of resuscitation by bystanders. Performance assessments are necessary to ensure that skills are acquired. We aimed to examine the validity of developed performance assessments and to determine credible pass/fail standards.

**Methods:**

Validity evidence was gathered in a standardised simulated setting by testing participants with three different levels of PBLS/FBAOM experience: untrained laypersons, trained laypersons, and lifeguards. Two blinded raters assessed participants’ performance. The reliability of test scores was analysed using generalizability theory, scores were compared across the three groups, and pass/fail-standards were established.

**Results:**

A total of 33 participants were included. More than two raters and two cases were necessary for PBLS to achieve a reliability coefficient above 0.80, which is considered the minimally acceptable level for high-stakes certification. For FBAOM, two tests or three raters were needed. Assessment scores differed across the three groups for PBLS skills, as well as for FBAOM skills (*p* < 0.001).

Pass levels of 74% and 55% of the maximum score for PBLS and FBAOM, respectively, were identified as the levels that best discriminated between competent and non-competent laypersons.

**Conclusions:**

Laypersons’ PBLS and FBAOM skills can be assessed in a reliable and valid way in a standardised simulated setting. However, multiple raters and scenario tests are needed to ensure sufficient reliability, which raises questions regarding the feasibility of performing certification tests for laypersons who participate in short paediatric resuscitation courses.

**Electronic supplementary material:**

The online version of this article (10.1186/s13049-018-0544-8) contains supplementary material, which is available to authorized users.

## Background

Survival from out-of-hospital paediatric cardiac arrest depends on fast recognition and initiation of resuscitation by bystanders [1–3]. To increase paediatric survival, relevant target groups, including daycare employees and other non-medical personnel working with children, need to possess resuscitation skills. Standardised courses for laypeople in Paediatric Basic Life Support (PBLS) and Foreign Body Airway Obstruction Management (FBAOM) are designed to teach the necessary skills based on international guidelines [4]. However, assessments are needed to ensure that course participants have acquired the skills necessary to deliver effective PBLS and FBAOM in the future.

Existing assessment instruments for paediatric resuscitation skills are directed at highly skilled health professionals who work in an in-hospital setting [5–7]. Effective first response intervention requires less advanced skills, than those expected in-hospital and can be taught to laypersons with no pre-existing medical training. Previous studies have used assessment instruments adapted from guidelines or extrapolated from existing assessment instruments designed for resuscitation of adults to determine readiness for practice [8–11]. However, such assessments may not be valid markers of competence when used for different populations, skills, and purposes [12].

Assessment of laypersons’ PBLS and FBAOM skills should have established validity evidence to support the interpretations made based on the assessment scores (i.e. is this person able to deliver effective PBLS/FBAOM?). In a recent study, essential items for the assessment of the two lifesaving skills, PBLS and FBAOM, were identified in an international consensus study [13]. However, evidence supporting the interpretation of test scores based on these items needs to be established. Without established validity evidence the value of assessments for both formative (e.g. assessment for feedback) and summative purposes (e.g. assessment for certification) is limited [14–16].

The objectives of this study were to collect validity evidence for the assessment of laypersons’ PBLS and FBAOM skills and to establish credible pass/fail standards.

## Methods

### Study design and setting

The study was conducted in a simulated setting in Copenhagen, Denmark and enrolled 33 laypersons between March and June 2017.

The study was deemed exempt from ethics approval by the Ethical Committee of the Capital Region, Copenhagen Denmark (Protocol no. 17006007). The Danish Data Protection Agency approved the study (j.nr: 2012–58-0004). All participants provided informed consent prior to enrolment in the study.

Messick’s framework for validity evidence was used in this study and is recommended by the American Education Research Association and the American Psychological Association in the 2014 *Standards for Educational Testing* [17]. The framework includes five categories of evidence: content, response process, internal structure, relation to other variables, and consequences [12]. A flowchart depicting the categories and the study design used to collect evidence is available in the appendix (Additional file 1 - Appendix figure 1).

### Participants

Purposive and convenience sampling was strategically performed to include three different groups: untrained laypersons, laypersons trained on PBLS and FBAOM, and lifeguards.

The three participant groups included in this study represented different levels of PBLS/FBAOM experience and were expected to have increasing levels of PBLS/FBAOM skills.

The untrained laypersons were daycare employees with no resuscitation training in the past year.

The trained laypersons group consisted of daycare employees, who participated in a two-hour hands-on standardised instructor-led course with up to six participants, immediately prior to the scenario tests. The course involved focused training on child and infant PBLS and FBAOM skills following ERC guidelines [4] and used the same manikins as the PBLS and FBAOM scenario tests. Instructors were basic life support certified instructors with additional paediatric training.

Lifeguards participated in a three-day intensive course just prior to the scenario tests. The course involved general first aid and basic life support provider resuscitation training with additional resuscitation training for children and infants.

Exclusion criteria for untrained and trained laypersons were any first aid training within one year, any type of health professional education. Skills generally decay over as little as six months and we chose a minimum of one year to avoid influence from previous training [7].

### Performance tests

**Fig. 1:**
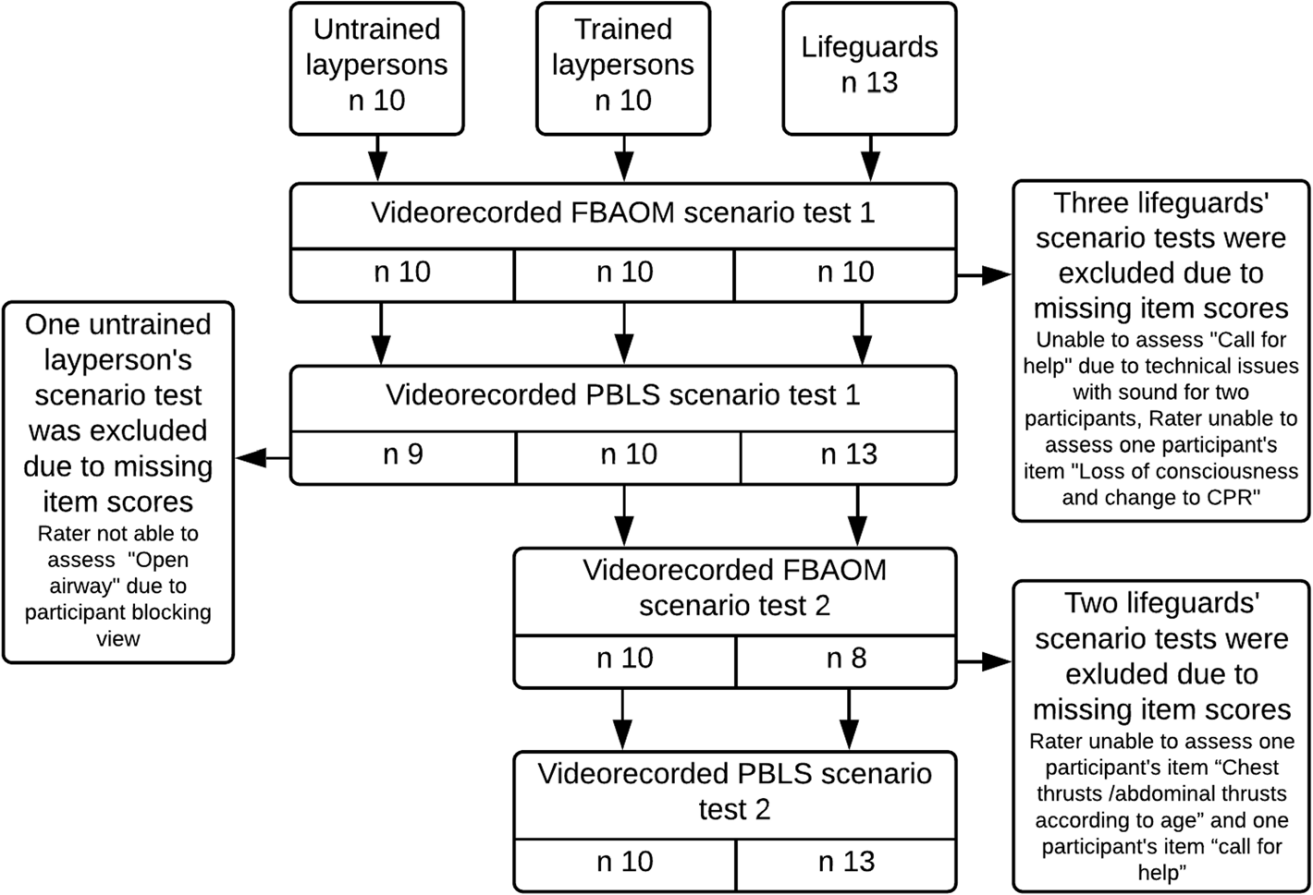
Flowchart for tests by participants. Legend to Fig. 1: The flowchart illustrates the participants in the scenario tests by group and exclusions due to missing rater scorings

The participants conducted two standardised simulated scenario tests for PBLS and FBAOM, respectively (Fig. 1).

Prior to the testing, participants were introduced to the simulated environment and informed about the purpose of the tests. A test facilitator led the scenarios using a standardised instruction protocol.

The PBLS scenario test included a child who was found lifeless on the floor in a daycare. The participant was alone at the scene and a helper was present elsewhere in the daycare centre. The PBLS test was conducted using Little Junior™ manikins (Laerdal Medical, Stavanger, Norway). The FBAOM test scenario involved an infant with sudden foreign body airway obstruction with rapid deterioration into unconsciousness. The Baby Anne™ manikin (Laerdal Medical, Stavanger, Norway) was used for the FBAOM tests. The scenario context was explained to the participants*:* E.g. “*You are alone in a daycare centre with a ten month old child who suddenly gets something stuck in the throat. The child is coughing loudly, awake and crying. There is no one else nearby. Show what you would do.”*

The scenario tests were repeated once with slight alterations in the child’s age and circumstances (Fig. 1). The clinical problem was identical for the two repeated tests and the expected actions according to current guidelines were the same.

Each test had a duration of approximately two to five minutes. The tests were video-recorded and viewed using iPads™ (Apple, California, USA).

The *content* of the PBLS and FBAOM assessment instruments was determined in an international Delphi consensus study which identified which elements should be included in assessments of laypersons [13]. The instruments included nine items for PBLS and eight for FBAOM. One item for PBLS “Use of AED” was not applicable for the training of the layperson group and hence excluded. Each assessment item was evaluated based on five-point scales. The research group developed descriptive anchors for values one, three and five, which targeted expectations for laypersons. The authors discussed the descriptive anchors until consensus was achieved.

Five-point scales were used instead of checklists to better capture increasing levels of competence [18].

The resulting assessment instruments for PBLS and FBAOM are shown in the appendix (Additional file 1 – Appendix tables 1 and 2).

A pilot test revealed that four out of eight FBAOM items could be assessed based on video-recorded scenario tests, and that for one FBAOM item (“Identify loss of consciousness and change to CPR”) only part of the original item could be assessed. The ability to identify unconsciousness was not possible to assess due to the limitations imposed by the manikin, and consequently, only the participant’s actions in response to unconsciousness were assessed.

The individual item scores were added to generate an assessment score. The maximum score for the two instruments were 40 and 20 points for PBLS and FBAOM, respectively. In addition to the item scores, the scenario tests were assessed using a 7-point global rating scale for the participant’s performance (1 = poor – 7 = excellent).

The *response process* included assessment of the scenario test videos in a random order by two blinded raters, who were European Resuscitation Council (ERC) certified BLS instructors. The raters participated in a 5-h rater-training course prior to rating the scenario tests. During the rater-training course, pilot rating videos were assessed and discussed with raters until consensus was reached.

### Statistics

The *internal structure* was examined by Generalizability (G) theory to examine the variances that influenced the reliability of the PBLS and FBAOM assessment scores.

G theory allows analysis of all the sources of variance (facets) and their interactions at the same time, such as interrater and test-retest variance, and enables the prediction of how test reliability changes when facet conditions are changed [19]. G theory is recommended for producing reliability estimates when assessing procedural skills [20].

The assessment scores of trained laypersons and lifeguards by each of the two raters were analysed separately for FBAOM and PBLS. The analysis was done using the G1 G theory program for SPSS [21]. Untrained laypersons were not included, as they are not the intended target population for the assessment instruments, and would, therefore, overinflate the reliability coefficients without reflecting the test’s intended use [22]. We used a fully-crossed two-facet design, with raters and tests as facets to estimate variances from these sources.

The variance attributed to the participants was considered the true variance reflecting different levels of competence. Error contributions were variances that related to raters and tests, as well as interactions with these. The percentage of the total variance was calculated to explain the true score fraction of the PBLS and FBAOM scores, respectively. Subsequently, the variance components were used in a decision-study (d-study) to determine the number of tests and raters needed to provide reliable judgments. A G coefficient of 0.8 is generally considered sufficient for high-stakes exams and 0.6 sufficient for formative feedback [19].

Internal consistency was examined using Cronbach’s alpha for the PBLS and FBAOM assessment instrument items, separately. Correlations of assessment instrument scores and global rating scores were analysed using Pearson’s correlation coefficients.

The *relationship to other variables* was examined by group comparisons. Assessment scores were the mean of the two raters’ scores as a percentage of maximum score. The assessment scores were compared using one-way analysis of variances (ANOVA) across the three groups and Bonferroni post hoc analysis between groups to examine their abilities to discriminate between different levels of skill. Only the assessment scores for the first scenario test for PBLS and FBAOM were included to avoid a testing effect [23].

The *consequences* were examined by the contrasting groups’ method to determine a pass/fail level based on the distribution of mean scores for untrained laypersons and lifeguards [24].

The intersection of the score distribution for the two groups indicated the level which ensures as few false negatives (failing competent performers - lifeguards) and false positives (passing incompetent performers – untrained laypersons) as possible. The contrasting groups’ pass/fail level and theoretical false positive and false negative distributions were calculated using a previously published Excel code [24].

SPSS version 24 was used for all other statistical analyses. A significance level of 0.05 was used for all analyses.

## Results

Characteristics of the participants are shown in Table 1. There were six missing assessment scores out of a total of 112 possible assessment scores (Fig. 1).

**Table 1:** Characteristics of participants

	Untrained laypersons	Trained laypersons	Lifeguards
Participants (n)	10	10	13
Sex, female	10 (100)	10 (100)	9 (69)
Age, years	40 (34–46)	49 (35–53)	20 (20–21)
Working with children, Years	10 (3–12)	11 (7–25)	–
No prior training	3 (30)	3 (30)	–
Years since training			
2–5	1 (10)	3 (30)	–
> 5	6 (60)	4 (40)	–

Continuous data are reported as median (Interquartile range). Dichotomous data reported as n (%)

Table 2 demonstrates results from the validation process structured according to Messick’s five sources of validity evidence.

**Table 2:** Validity evidence results by category

Validity evidence source	Question related to the source of evidence	Validity evidence for the assessment	
		PBLS	FBAOM
Content	Is the content measuring the intended construct (skill levels of laypersons)?	International resuscitation experts identified the assessment items as essential for laypersons	
Response process	Are bias sources reduced?	One item was not applicable to the layperson training and excluded.	Pilot testing of the rating procedure revealed 4 FBAOM items could not be scored.
		The raters participated in rater training and participants’ skill levels were blinded for the raters.	
Internal structure	Are the test scores reliable?	The generalizability analysis and the d-study identified the number of tests and raters needed for different levels of reliability.	
		Pearson’s correlations above 0.93 (*p* < 0.001) between global ratings scores and assessment scores support the construct of the test.	
		The high Cronbach’s alpha supports the match of items and the intended construct	The questionable Cronbach’s alpha suggests internal inconsistency in the test items.
Relation to other variables	Does the score correlate with other measures of skills?	The assessment scores increased with increasing duration of training and significantly differentiated all the three groups.	The assessment scores increased with training and discriminated untrained laypersons from all other groups. The assessment scores were not able to discriminate trained laypersons from lifeguards.
Consequences	What is the consequences of the pass/fail score	All untrained laypersons and one lifeguard failed. Theoretical false positives and negative with the contrasting groups method was 1.0% and 0.5%, respectively.	Eight untrained laypersons and three lifeguards failed. Theoretical false positives and negative with the contrasting groups method was 22% and 29%, respectively
		Unintended consequences of the pass score could be low self-efficacy and reluctance to intervene in real resuscitation attempts	

The table shows the five categories and the validity evidence in each category

**Fig. 2:**
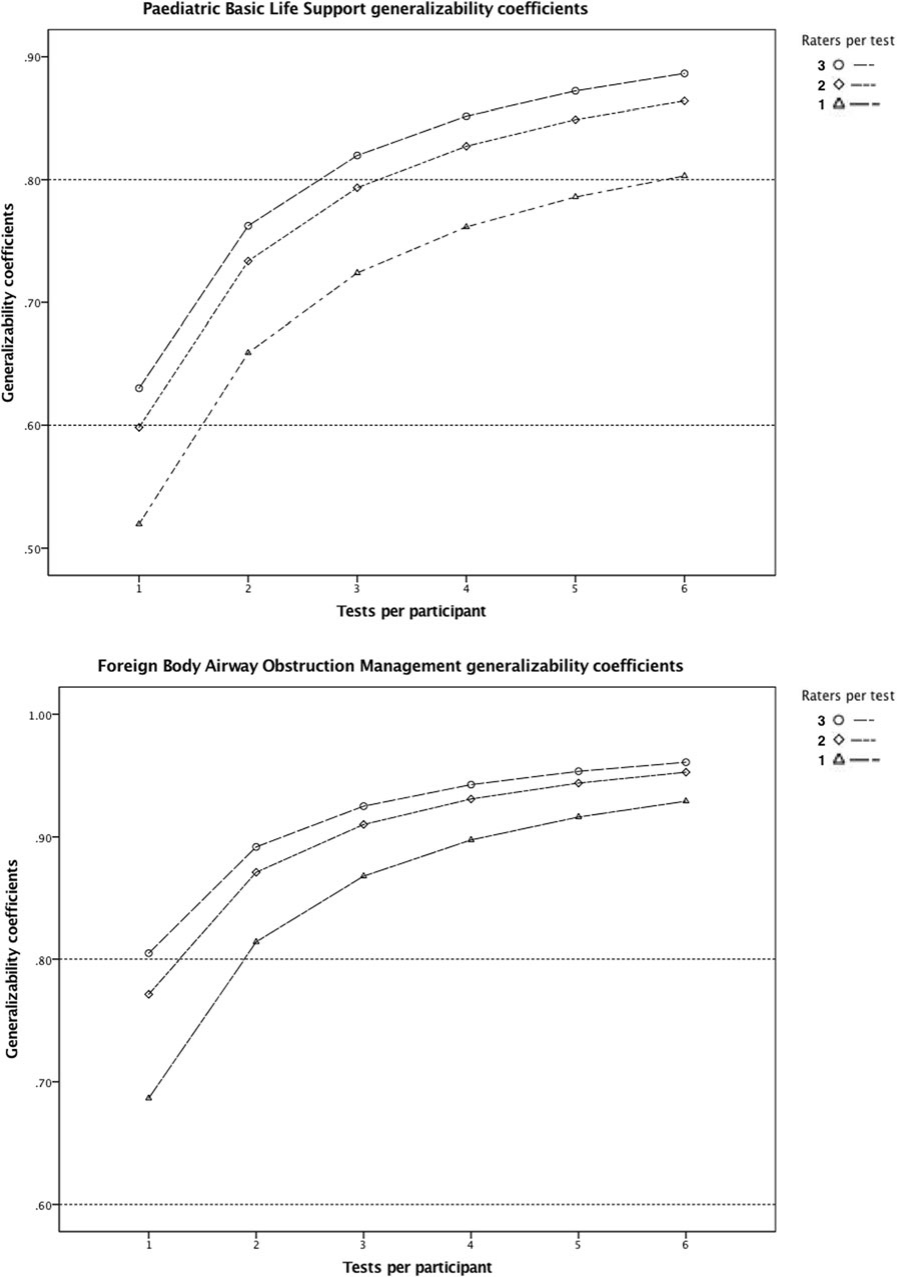
Paediatric Basic Life Support and Foreign Body Airway Obstruction Management d-study results. Legend Fig. 2: The graphs illustrate the generalizability coefficients for different numbers of raters and tests per participant for Paediatric Basic Life Support and Foreign Body Airway Obstruction Management. The lines at 0.6 and 0.8 represent the level needed for formative feedback and certification, respectively

The generalizability analysis is shown in appendix (Additional file 1 – Appendix table 4). The d-study results are shown in Fig. 2. The d-study demonstrated that three raters and three cases or one rater and six cases were needed to achieve a reliability coefficient of 0.80 for PBLS. For FBAOM, three raters or two tests were needed. The Cronbach’s alpha was 0.94 and 0.64 for PBLS and FBAOM assessment item scores, respectively. Pearson’s correlation coefficients between the assessment scores and the global rating scores were r(30) = 0.93, *p* < 0.001 for PBLS and r(28) = 0.96, p < 0.001 for FBAOM.

PBLS and FBAOM assessment scores differed significantly across the three groups for both PBLS (F(2,29) = 64.01, p < 0.001) and FBAOM (F(2,27) = 13.04, p < 0.001). Mean scores and post-hoc analysis are shown in Table 3.

**Table 3:** Assessment score means and post hoc analysis

	Untrained laypersons, mean (95% CI)	Trained laypersons, mean (95% CI)	Lifeguards, mean (95% CI)	One-way-ANOVA	Post hoc analysis (t-test with Bonferroni corrected *p*-value*)		
					Untrained laypersons vs. Trained laypersons	Untrained laypersons vs. lifeguards	Trained laypersons vs. lifeguards
PBLS	47.08 (38.41–55.76)	78.63 (72.06–85.19)	89.62 (85.85–93.38)	F(2,29) = 64.011, *p* < 0.001)	t(18) = −5.21, *p* < 0.001	t(20) = −11.38, *p* < 0.001	t(21) = −3.42, *p* = 0.02
FBAOM	46.50 (38.54–54.46)	75.00 (65.54–84.46)	62.25 (52.92–71.58)	F(2,27) = 13.038, *p* < 0.001	t(17) = −6.72, *p* < 0.001	t(18) = −2.91, *p* = 0.03	t(18) = 2.17, *p* = 0.09

The table shows the ANOVA of assessment mean scores as per cent of max score for PBLS and FBAOM and the Bonferroni adjusted post hoc analysis. *Values < 0.05 are considered significant

The individual item scores and analysis are presented in the appendix (Additional file 1 – Individual item scores).

**Fig. 3:**
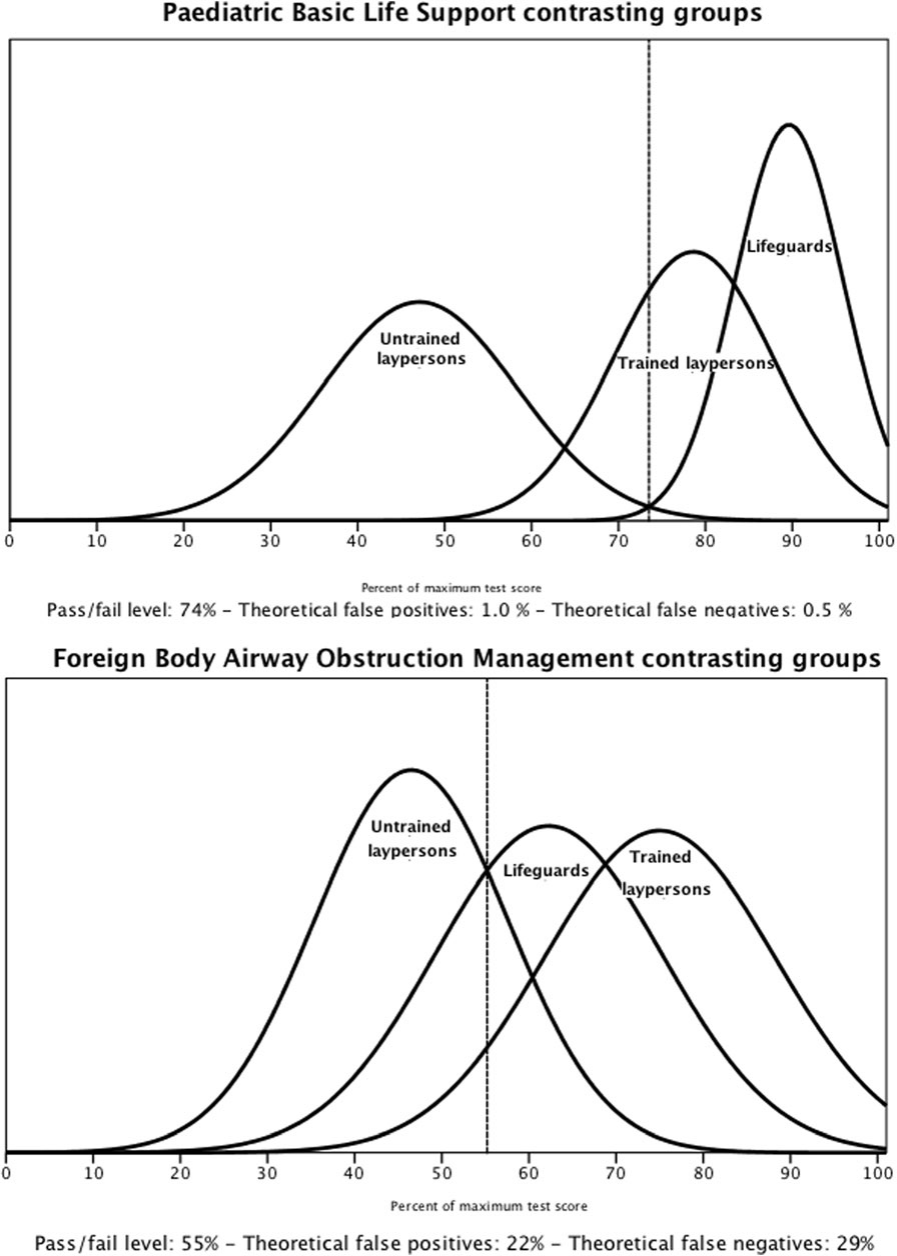
Paediatric Basic Life Support and Foreign Body Airway Obstruction Management contrasting groups. Legend Fig. 3: The figures illustrate the distributions of the three groups and the pass/fail level for Paediatric Basic Life Support and Foreign Body Airway Obstruction Management based on the intersection between the untrained laypersons’ and lifeguards’ distributions. The theoretical distributions of false positives and false negatives are displayed. The Y-axis illustrates the relative number of participants receiving each score

The pass/fail level was established as 74% and 55% of the maximum score for PBLS and FBAOM, respectively (Fig. 3). All the untrained laypersons, 20% of the trained laypersons and 8% of the lifeguards failed the PBLS scenario test. For FBAOM, 80% of the untrained laypersons, none of the trained laypersons and 30% of the lifeguards failed.

## Discussion

The validity evidence supports the assumption that increasing scores reflect increasing levels of PBLS and FBAOM skills. The PBLS and FBAOM assessment scores significantly discriminated untrained from trained laypersons and lifeguards (Table 3). The validity argument apparent in our findings is further supported by the strong correlations between PBLS/FBAOM assessment scores and the global rating scores.

The PBLS d-study (Fig. 2) shows that two tests or two raters are needed to reach G coefficients of 0.6 which are sufficient for formative feedback, and six tests for one rater or three tests and two raters are needed for high stakes certification G coefficients of 0.8. For FBAOM (Fig. 2), a G coefficient of 0.6 requires one test and one rater, and a G coefficient of 0.8 requires at least two tests or three raters.

A generalizability analysis for residents’ advanced paediatric life support skills found similar results such that additional tests increased reliability more than additional raters [5]. In fact, 12 tests were needed for a generalizability coefficient of 0.73, and another study with ten tests and two raters resulted in a G coefficient of 0.94 [6].

The results of our d-study reflect the need for fewer tests to reach sufficient reliability. This may be because our scenario tests were less specialised, and test the same skills in each scenario test, as illustrated by the very low variance contribution from tests in the g study (Additional file 1 – Appendix table 4).

Certification of layperson may not be feasible within the short duration of traditional PBLS courses without compromising the time dedicated to actual PBLS training. However, reliability coefficients sufficient for formative feedback to improve learning may be achievable for both PBLS and FBAOM [16]. In addition, the process of testing individuals could also, by itself, induce a learning effect [23].

FBAOM assessment scores revealed that the lifeguards, who were expected to perform at the highest level, were matched by trained laypersons (Table 3). The trained laypersons participated in specific FBAOM training just prior to the scenario test. In addition, the infant FBAOM skills may be mostly relevant for daycare employees which may increase motivation among laypersons to learn these skills, whereas the lifeguards may be more focused on skills that they are expected to master, such as FBAOM for adults and general resuscitation skills. The findings are similar to a previous assessment of residents in paediatric advanced life support, where experience did not affect performance, but specific training improved all residents’ performance [6]. An alternative explanation is that the assessment instrument was not able to capture experts’ skills, which may rely on shortcuts and less strict adherence to a step-by-step approach than the approaches of untrained laypersons [25]. However, the high correlation with the overall performance score of 0.96 suggests that this was not the case.

For PBLS, the pass/fail level of 74% clearly discriminated competent from non-competent performers and the theoretical distributions revealed only 1.0% false positives (passing incompetent performers) and 0.5% false negatives (failing competent performers) (Fig. 3).

For FBAOM, the pass/fail level was 55% and the theoretical distribution of scores resulted in 22% false positives (passing incompetent performers) and 29% false negatives (failing competent performers) (Fig. 3).

Most untrained laypersons can attain sufficient skill levels with short standardised training for both PBLS and FBAOM (Fig. 3). Performance improvements has also been demonstrated for laypersons who receive brief training in adult resuscitation skills [26, 27].

However, the pass/fail level for FBAOM allows a large proportion of non-competent performers to receive a passing score. Hence, the level may not be advisable for the purpose of certification, particularly given the low reliability if only a single test and a single rater are used. Moreover, there may be unintended consequences of failing some course participants with respect to reduced self-efficacy and willingness to initiate real resuscitation attempts, which in turn, may reduce the chance of survival [1–3]. On the other hand, passing a course implies that participants have attained certain skills which can be used to provide effective resuscitation attempts.

The reliability results are strengthened by inclusion of only trained laypersons and lifeguards in the generalizability analysis, as reliability indices will be artificially overinflated by including complete untrained in the calculation [22, 28].

A limitation to the study is the number of participants, although the sample size was larger than the median sample size (*n* = 25) of education research studies [29], and significant differences were identified between groups.

We used convenience sampling which may have resulted in selection of participants who were more motivated about training than the general population. In turn, this may have resulted in better performance among untrained and trained laypersons. However, we believe that most daycare workers are motivated about gaining paediatric resuscitation skills.

Internal consistency of the FBAOM test was questionable (Cronbach’s alpha = 0.64). One item “call for help” seemed to be problematic (Additional file 1 - Appendix table 3). The item failed to discriminate between groups (F(2,28) = 2.27, *p* = 0.12) and omitting it from the FBAOM assessment instrument may be advisable from a psychometric point of view, as it does not help to discriminate between the three groups of performers. However, content experts considered this item essential for the assessment [13] and it is still a vital part of the chain of survival [30]. For these reasons, we chose to retain the item, as we suspect that the poor fit in our study reflects failure to assess participants’ ability to call for help in the simulated setting rather than that the item is non-essential.

The primary implication of the study is that the PBLS and FBAOM assessment instruments can be used to assess laypersons’ PBLS and FBAOM skill levels. The assessment scores make it possible to compare outcomes from different training methods and to assess the quality of various courses. Moreover, the use of standardised performance standards enables competency-based training as an alternative to current time-based models.

The reliability analyses suggest that the assessment instruments can be used for formative feedback to increase learning for laypersons, but not for summative certification purposes if only one or two tests administered. However, if certification of laypersons skills is needed courses should be designed with additional time to allow for an appropriate number of tests and raters for defensible certification of skill levels.

## Conclusions

The study found evidence to support the use of standardised assessment instruments to measure increasing skill levels in PBLS and FBAOM.

Reliable assessments of performance for formative feedback purposes are attainable. However, multiple raters and scenario tests are needed to ensure reliability which is sufficient to justify PBLS and FBAOM certification, and this may not be feasible during brief training courses for laypersons.

## Additional file

Additional file 1: The appendix includes an overview of the scoring instruments, flowchart for collecting validity evidence, analysis of individual item scores and results of the generalizability analysis. (PDF 626 kb)

## Abbreviations

ERC: European Resuscitation Council
FBAOM: Foreign Body Airway Obstruction Management
PBLS: Paediatric Basic Life Support
