# Supplementary material for: Assessment of laypersons’ paediatric basic life support and foreign body airway obstruction management skills: a validity study
Source: Scand J Trauma Resusc Emerg Med. 2018 Sep 6;26:73. doi: 10.1186/s13049-018-0544-8 (PMC6127933; doi:10.1186/s13049-018-0544-8)
Supplement: Supplementary file 1 — The appendix includes an overview of the scoring instruments, flowchart for collecting validity evidence, analysis of individual item scores and results of the generalizability analysis. (PDF 626 kb) [file 13049_2018_544_MOESM1_ESM.pdf]

Appendix

Scoring instruments

Appendix table 1 – Paediatric Basic Life Support assessment instrument

The greyed-out *italic* item is not applicable in the video-recorded scenario tests

PBLS	Item description	Likert scale				
		1	2	3	4	5
**Responsiveness**	Recognise unresponsiveness	No attempt to recognise unresponsiveness	-	Recognises unresponsiveness but inefficiently	-	Efficiently recognises unresponsiveness
**Call for help**	Recognise need for help and alert surroundings both by loud verbal call out and using telephone	No attempt to call for help	-	Calls for help but not done efficiently	-	Efficiently calls for help
**Open airway**	Establish open airways including mouth inspection, appropriate head and jaw positioning.	No attempt to open airway	-	Establishes open airway but inappropriately	-	Appropriately establishes open airway

**Check breathing**	Assess breathing and recognise respiratory arrest or abnormal breathing	No attempt to assess breathing	-	Assesses breathing but inefficiently	-	Efficiently assesses breathing and recognises respiratory arrest or abnormal breathing
**Rescue breaths**	Provide high quality initial rescue breaths	No attempt to provide rescue breaths	-	Delivers some effective rescue breaths	-	Consistently provides effective rescue breaths
**Compressions**	Provide high quality compressions. Adequate rate, compression depth and correct hand placement	No attempt to provide compressions	-	Provides some high quality compressions	-	Consistently provides high quality compressions
**Ventilations**	Provide high quality ventilations in general during CPR with adequate chest rise.	No attempt to provide ventilations	-	Provides some effective ventilations	-	Consistently provides effective ventilations
**Time factor**	Act effectively	No optimal use of	-	Optimal time use	-	Consistently

	with minimised hands-off time, no delays in treatment and fast call for help	time		inconsistently		optimal time use
Use of AED	*Call for Automatic External defibrillator and appropriate use*	*No attempt to call for AED*	-	*Calls for AED but used inadequately*	-	*Adequate use of AED*

Appendix table 2 Foreign Body Airway Obstruction Management assessment instrument

The greyed-out *italic* items are not applicable in the video-recorded scenario tests.

FBAOM scoring	Item description	Likert scale				
		1	2	3	4	5
Identify different stages of foreign body airway obstruction	*Distinguish effective and ineffective cough*	*No attempt to distinguish cough*	*-*	*Inconsistently distinguishes effective and ineffective coughs*	*-*	*Consistently distinguishes effective and ineffective coughs*
Identify consciousness	*Recognise unresponsiveness*	*No attempt to recognise responsiveness*	*-*	*Recognises responsiveness but inefficiently*	*-*	*Efficiently recognises responsiveness*
**Call for help**	Recognise need for help and alert surroundings	No attempt to call for help	-	Calls for help but not done efficiently	-	Efficiently calls for help
**Back blows**	Provide high quality back blows with adequate force and correct placement of impact	No attempt to provide back blows	-	Provides some high quality back blows	-	Consistently provides high quality back blows
**Chest thrusts**	Provide high	No attempt to	-	Provides some high	-	Consistently

**/abdominal thrusts according to age**	quality chest thrust or abdominal thrust according to age	provide thrusts		quality thrusts		provides high quality thrusts
**Identify loss of consciousness and change to CPR**	Recognise changes in condition and act appropriately	No reaction to loss of consciousness	-	Reacts appropriately but inefficiently	-	Reacts appropriately and efficiently
**Assessment of breathing**	Assess breathing and recognise respiratory arrest or abnormal breathing requiring ventilator support	No attempt to assess breathing	-	Assesses breathing but inefficiently	-	Efficiently assesses breathing and recognises respiratory arrest or abnormal breathing
**Ventilation**	Provide high quality ventilations if patient stops breathing with adequate chest rise	No attempt to provide ventilations	-	Provides some effective ventilations	-	Consistently provides effective ventilations

Flowchart for collecting validity evidence

Appendix figure 1: Flowchart for collecting validity evidence

The flowchart describes the five sources of validity evidence and the study design to collect evidence in the five categories.

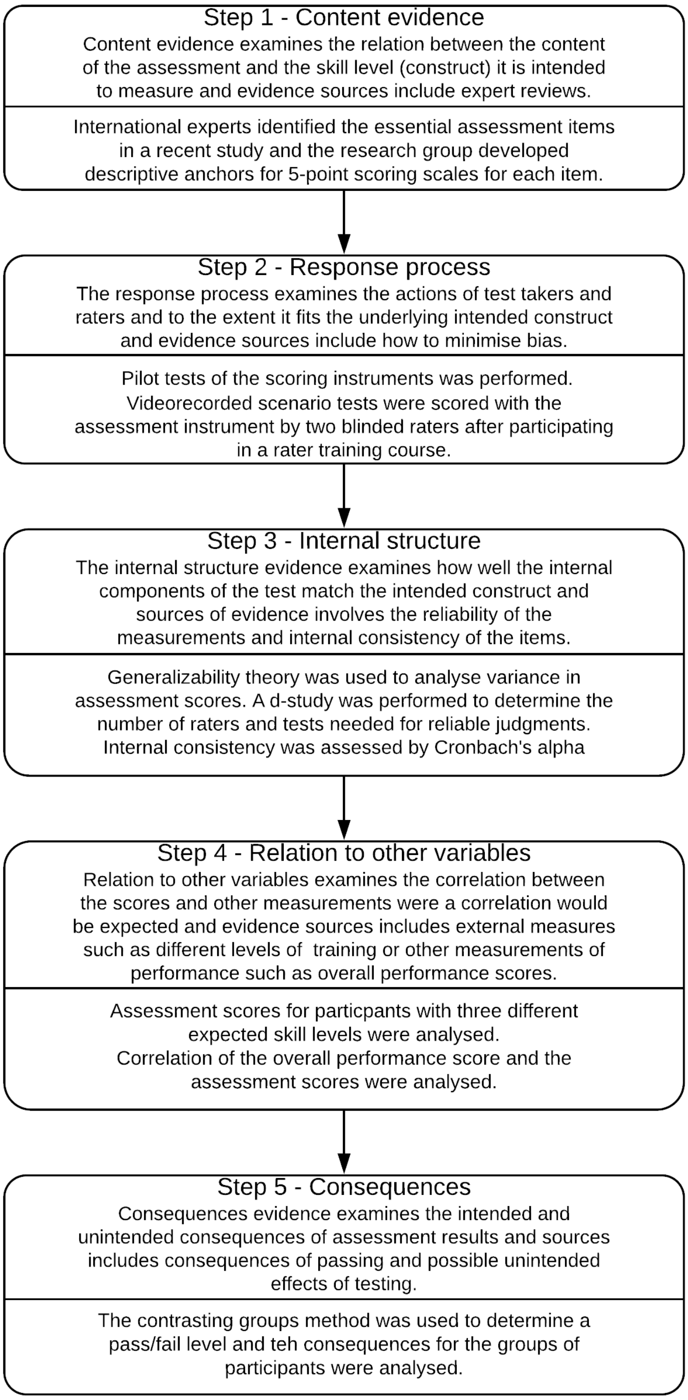

Individual item scores

The figures (appendix figure 2 and 3) portrait the mean individual item scores by each group. The mean individual item scores for PBLS increased for all items from untrained to trained laypersons to lifeguards.

ANOVA and post hoc analysis of individual items for both PBLS and FBAOM items across the three groups is visible in appendix table 3. The ANOVA was significant for all PBLS and FBAOM items except the FBAOM item "Call for help".

In post hoc analysis for PBLS all items but "Call for help" differed from untrained to trained laypersons. There were no statistically significant differences between trained laypersons and lifeguards. "Call for help" was significantly higher in the lifeguard group compared with the untrained layperson group ($t(21)=-3.27$ $p=0.008$)

For the FBAOM test "Call for help" was non-significant in an one-way ANOVA across the three groups ($F(2,28) = 2.27$, $p=0.12$). The post hoc analysis found significant differences between untrained and trained laypersons' scores for "backblows" ($t(18)=-5.40$ $p<0.001$), trained laypersons and lifeguards ($t(21)=3.76$ $p=0.003$) and not between intermediates and lifeguards ($t(21)=-2.07$ $p=0.14$).

The FBAOM items "Chest thrusts "and "change to CPR" was significantly different between all three individual groups ($p<0.001$).

The internal consistency analysed by Cronbach's alpha standardised items were 0.94 for the PBLS eight item scale and 0.64 for the FBAOM four item scale. The ANOVA above identified the call for help score as non-significantly different and an analysis of the internal consistency when this item was left out resulted in a Cronbach's alpha of 0.93.

Appendix Figure 2 – Paediatric Basic Life Support scores for individual assessment items by group

The figure illustrates the mean scores with 95% confidence intervals for the eight Paediatric Basic Life Support items.

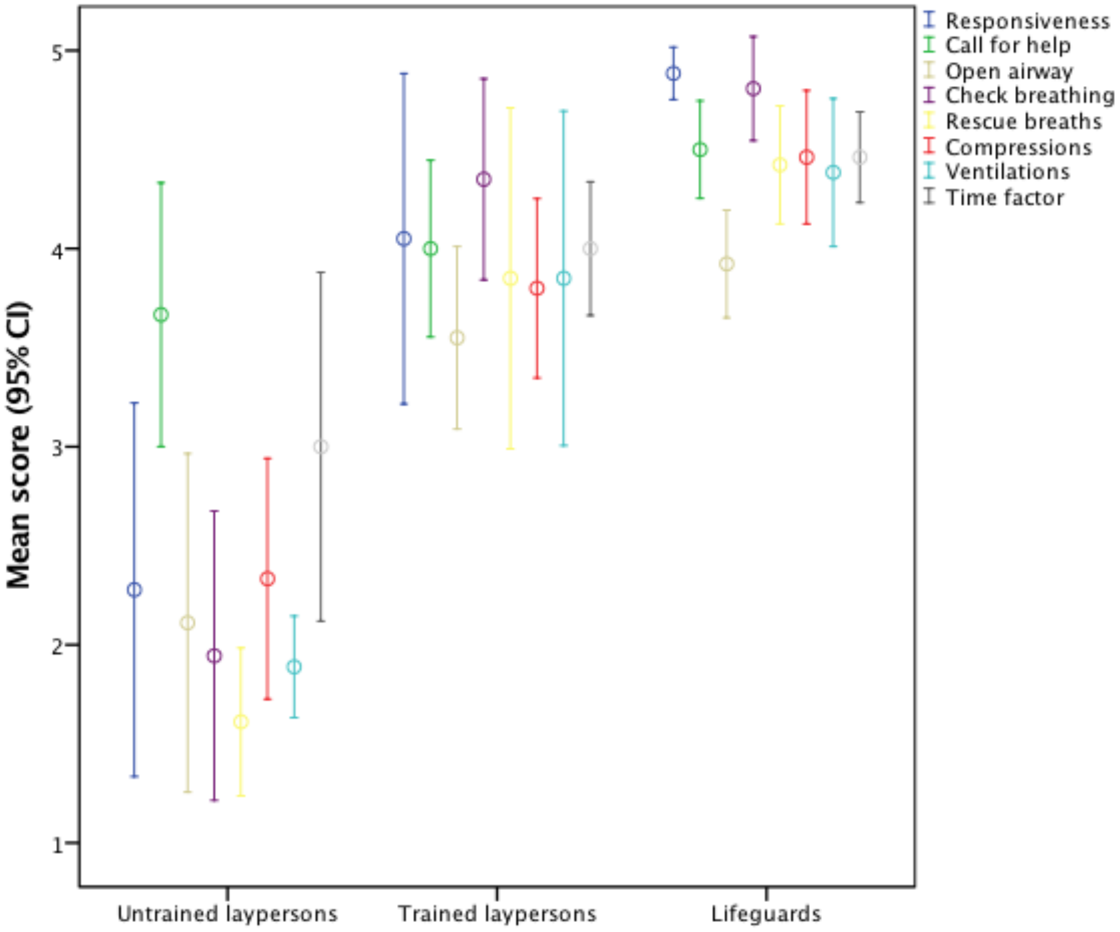

Appendix figure 3 – Foreign Body Airway Obstruction scores for individual assessment items by group

The figure illustrates the mean scores with 95% confidence intervals for the four Foreign Body Airway Obstruction Management items.

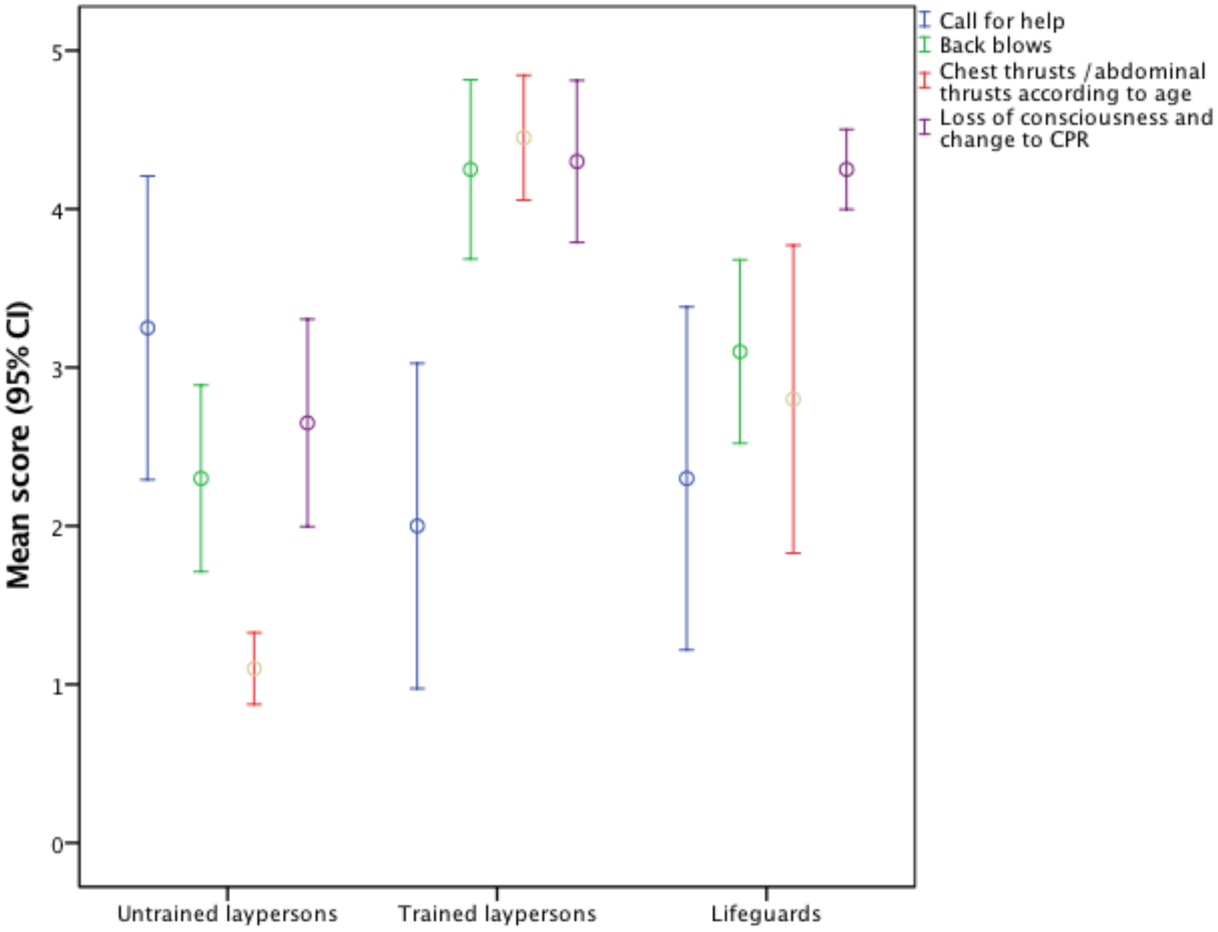

Appendix table 3

The table shows the results of individual items ANOVA and the post hoc Bonferroni corrected analysis for both Paediatric Basic Life Support and Foreign Body Airway Obstruction Management items.

**Item**	**Untrained laypersons, mean (95%CI)**	**Trained laypersons, mean (95%CI)**	**Lifeguards, mean (95%CI)**	**ANOVA**	**Post hoc analysis**		
					**Untrained vs. trained laypersons**	**Untrained laypersons vs. lifeguards**	**Trained laypersons vs. lifeguards**
**PBLS**							
Responsiveness	2.55 (1.51 - 3.58)	4.05 (3.21 - 4.88)	4.88 (4.75 - 5.02)	F(2,30) =14.77 p<0.001	t(18)=-2.56 p=0.01	t(21)=-5.79 p<0.001	t(21)=-2.54 p=0.19
Call for help	3.65 (3.06 - 4.23)	4 (3.55 - 4.44)	4.5 (4.25 - 4.75)	F(2,30) =5.47 p=0.009	t(18)=-1.08 p=0.65	t(21)=-3.27 p=0.008	t(21)=-2.32 p=0.19
Open airway	2.11 (1.25 - 2.96)	3.55 (3.08 - 4.01)	3.92 (3.65 - 4.19)	F(2,29) =16.63 p<0.001	t(17)=-3.5 p<0.001	t(20)=-5.33 p<0.001	t(21)=-1.64 p=0.73

Check breathing	1.85 (1.17 - 2.52)	4.35 (3.84 - 4.85)	4.81 (4.54 - 5.07)	F(2,30) =54.89 p<0.001	t(18)=-6.7 p<0.001	t(21)=-10.04 p<0.001	t(21)=-1.91 p=0.40
Rescue breaths	1.65 (1.31 - 1.98)	3.85 (2.98 - 4.71)	4.42 (4.12 - 4.72)	F(2,30) =38.60 p<0.001	t(18)=-5.38 p<0.001	t(21)=-13.58 p<0.001	t(21)=-1.56 p=0.27
Compressions	2.45 (1.85 - 3.04)	3.8 (3.34 - 4.25)	4.46 (4.12 - 4.8)	F(2,30) =25.66 p<0.001	t(18)=-4.09 p<0.001	t(21)=-6.95 p<0.001	t(21)=-2.66 p=0.08
Ventilations	1.9 (1.67 - 2.12)	3.85 (3.00 - 4.69)	4.38 (4.01 - 4.76)	F(2,30) =30.80 p<0.001	t(18)=-5.05 p<0.001	t(21)=-11.57 p<0.001	t(21)=-1.41 p=0.33
Time factor	3.05 (2.26 - 3.83)	4 (3.66 - 4.33)	4.46 (4.23 - 4.69)	F(2,30) =11.85 p<0.001	t(18)=-2.53 p=0.01	t(21)=-4.36 p<0.001	t(21)=-2.6 p=0.37
**FBAOM**							
Call for help	3.25 (2.29 -	2 (0.97 - 3.02)	2.18 (1.18 -	F(2,28) =2.27	t(18)=2.02 p=0.18	t(19)=1.72 p=0.29	t(19)=-0.29

	4.20)		3.18)	p=0.12			p>0.999
Back blows	2.3 (1.71 - 2.88)	4.25 (3.68 - 4.81)	3 (2.52 - 3.48)	F(2,30) =15.30 p<0.00 1	t(18)=-5.4 p<0.001	t(21)=- 2.07 p=0.14	t(21)=3. 76 p=0.003
Chest thrusts /abdominal thrusts according to age	1.1 (0.87 - 1.32)	4.45 (4.05 - 4.84)	2.92 (2.19 - 3.65)	F(2,30) =40.11 p<0.00 1	t(18)=- 16.69 p<0.001	t(21)=- 4.64 p<0.001	t(21)=3. 71 p<0.001
Loss of consciousne ss and change to CPR	1.1 (0.87 - 1.32)	4.45 (4.05 - 4.84)	2.92 (2.19 - 3.65)	F(2,29) =15.80 p<0.00 1	t(18)=-4.5 p<0.001	t(20)=- 4.63 p<0.001	t(20)=0. 83 p<0.001

Results of generalizability analysis

Appendix table 4

The table shows the variance components of the different facets and interactions; and relative contribution to the overall variance for Paediatric Basic Life Support and Foreign Body Airway Obstruction. In addition, an interpretation of the results from the different components is provided.

**Source of variance**	**Description**	**Paediatric Basic Life Support**			**Foreign Body airway obstruction**		
		**Variance components**	**Relative contribution (%)**	**Interpretation**	**Variance components**	**Relative contribution (%)**	**Interpretation**
**Participants**	Systematic variance between participants	57.874	48.3	Most of the variance is related to intended differences between participants	148.407	61.4	Most of the variance is related to intended differences between participants
**Tests**	Systematic variance among tests	0	0	Cases were equally difficult	2.124	0.9	Cases were almost equally difficult
**Raters**	Systematic variance among raters	8.362	7	A minor fraction of the variance was due to differences between raters	23.529	9.7	A minor fraction of the variance was due to differences between raters
**Interact**	Trend for	24.154	20.2	The tests	20.098	8.30	The tests

**ion between tests and participants**	tests to assess participants differently			varied in their assessment of participants			varied in their assessment of participants
**Interaction between raters and participants**	Trend for raters to assess participants differently	6.312	5.3	There was only minor bias between raters and participants due to effective blinding	0	0	There was no bias between raters and participants due to effective blinding
**Interaction between raters and tests**	Trend for rater to assess differently on cases	0	0	The raters found the cases equally challenging	0	0	The raters found the cases equally challenging
**Interaction between tests, raters and participants**	Residual variability and facets not included in the test design	23.116	19.3	Anticipated unexplained error	47.651	19.7	Anticipated unexplained error
